# Unraveling the Paradox: Can Anticoagulation Improve Outcomes in Patients With Heart Failure and Increased Bleeding Risk?

**DOI:** 10.7759/cureus.57544

**Published:** 2024-04-03

**Authors:** Danish Saeed, Daniel Fuenmayor, Jose A Niño Medina, Inam Saleh, Juan David Castiblanco Torres, Wendys L Horn, Mauricio H Sosa Quintanilla, Karen E Leiva, Valentina Dannuncio, Maria Viteri, Miguel Rivas, Neelam Kumari

**Affiliations:** 1 Internal Medicine, Shaikh Zayed Medical Complex, Lahore, PAK; 2 Surgery, Universidad de Carabobo, Valencia, VEN; 3 Faculty of Legal and Political Sciences, Universidad de Carabobo, Valencia, VEN; 4 Faculty of Health Sciences, Universidad de Carabobo, Valencia, VEN; 5 Pediatrics, University of Kentucky College of Medicine, Lexington, USA; 6 General Medicine, Antonio Nariño University, Bogota, COL; 7 School of Medicine, Universidad de El Salvador, San Salvador, SLV; 8 General Medicine and Surgery, National Autonomous University of Honduras, Tegucigalpa, HND; 9 Faculty of Medicine, National University of the South, Bahia Blanca, ARG; 10 Metabolic Syndrome Department, Hospital General Ambato, Ambato, ECU; 11 Internal Medicine, Jinnah Medical & Dental College, Karachi, PAK

**Keywords:** heart failure, diastolic heart failure, acute decompensated heart failure, heart failure with preserved ejection fraction, anticoagulant therapy, systolic heart failure

## Abstract

Heart failure (HF) patients frequently present with comorbidities such as atrial fibrillation (AF) or other cardiovascular conditions, elevating their risk of thromboembolic events. Consequently, anticoagulation therapy is often considered for thromboprophylaxis, although its initiation in HF patients is complicated by concomitant bleeding risk factors. This review explores the paradoxical relationship between HF, increased bleeding risk, and the potential benefits of anticoagulation. Through an examination of existing evidence from clinical trials, observational studies, and meta-analyses, we aim to elucidate the role of anticoagulation in HF patients with increased bleeding risk. Despite guidelines recommending anticoagulation for certain HF patients with AF or other thromboembolic risk factors, uncertainty persists regarding the optimal management strategy for those at heightened risk of bleeding. The review discusses the pathophysiological mechanisms linking HF and thrombosis, challenges in bleeding risk assessment, and strategies to minimize bleeding risk while optimizing thromboprophylaxis. Shared decision-making between clinicians and patients is emphasized as essential for individualized treatment plans that balance the potential benefits of anticoagulation against the risk of bleeding complications. Furthermore, it examines emerging anticoagulant agents and their potential role in HF management, highlighting the need for further research to delineate optimal management strategies and inform evidence-based practice. In conclusion, while anticoagulation holds promise for improving outcomes in HF patients, careful consideration of patient-specific factors and ongoing research efforts are essential to optimize therapeutic strategies in this population.

## Introduction and background

Heart failure (HF) poses a significant burden on global healthcare systems, affecting millions of individuals worldwide. Despite advances in treatment, HF remains a leading cause of morbidity and mortality [1]. Patients with HF often present with comorbidities such as atrial fibrillation (AF) or other cardiovascular conditions, which increase their risk of thromboembolic events. Consequently, the use of anticoagulants for thromboprophylaxis is common in this population. However, the decision to initiate anticoagulation therapy in HF patients is complicated by the presence of concomitant bleeding risk factors, including anticoagulant therapy, antiplatelet therapy, renal and liver dysfunction, older age, anemia, prior bleeding history, polypharmacy, falls/frailty, and the presence of comorbidities creating a clinical paradox [2]. While anticoagulation may reduce the risk of thromboembolic events, it may simultaneously increase the risk of bleeding complications, which can exacerbate HF symptoms and lead to adverse outcomes. The paradoxical relationship between HF, increased bleeding risk, and the potential benefits of anticoagulation is a topic of growing importance in clinical practice. Despite guidelines recommending anticoagulation for certain HF patients with AF or other thromboembolic risk factors, there is uncertainty regarding the optimal management strategy for those at increased risk of bleeding [3]. The complex interplay between thrombosis and bleeding in HF patients underscores the need for a comprehensive review of the existing evidence to guide clinical decision-making.

Therefore, the objective of this review is to examine the current evidence regarding the role of anticoagulation in patients with HF and increased bleeding risk. By synthesizing available data from clinical trials, observational studies, and meta-analyses, this review aims to elucidate the potential benefits and risks associated with anticoagulation therapy in this unique patient population. Ultimately, a better understanding of this complex clinical scenario is essential for optimizing patient outcomes and informing evidence-based practice in the management of HF. For this review, evidence was generated after thoroughly searching PubMed and Google Scholar, and relevant articles were separated.

## Review

Pathophysiology

HF is a complex syndrome characterized by the inability of the heart to pump blood effectively to meet the body's metabolic demands [4]. It can result from various etiologies, including myocardial infarction, hypertension, valvular heart disease, and cardiomyopathies. The pathophysiology of HF involves a cascade of neurohormonal activation, myocardial remodeling, and cellular dysfunction, leading to impaired cardiac contractility and relaxation [5]. One key aspect of HF pathophysiology relevant to thrombosis is the development of endothelial dysfunction. Endothelial cells play a crucial role in maintaining vascular homeostasis by regulating vasodilation, inflammation, and thrombosis. In HF, systemic and pulmonary endothelial dysfunction occurs due to increased oxidative stress, inflammation, and reduced nitric oxide bioavailability. This endothelial dysfunction promotes a prothrombotic state by impairing vasodilation, enhancing platelet activation, and increasing the expression of procoagulant factors [6].

Moreover, HF leads to the activation of the coagulation cascade through multiple mechanisms. The release of proinflammatory cytokines and neurohormones, such as angiotensin II and catecholamines, stimulates the production of tissue factors and other procoagulant molecules [7]. Additionally, stasis of blood flow in dilated cardiac chambers and reduced cardiac output contribute to blood stagnation, further predisposing to thrombus formation. Furthermore, HF-induced hemodynamic changes, such as increased atrial pressure in patients with HF with preserved ejection fraction (HFpEF) or AF, promote blood stasis in the atria, fostering thrombus formation [4]. The interplay between impaired cardiac function, endothelial dysfunction, and activation of coagulation pathways in HF creates a prothrombotic milieu, increasing the risk of thromboembolic events. Thrombotic complications in HF predominantly manifest as strokes or systemic embolisms, which can have devastating consequences for patient morbidity and mortality. The risk of thromboembolic events is particularly elevated in HF patients with comorbidities such as AF, which is commonly associated with HF and further exacerbates the prothrombotic state through atrial stasis and thrombus formation [2].

Overall, understanding the pathophysiological mechanisms linking HF and thrombosis is crucial for recognizing the heightened thromboembolic risk in HF patients and informing appropriate thromboprophylaxis strategies to mitigate adverse outcomes.

Bleeding risk assessment

Assessing bleeding risk in patients with HF poses several challenges due to the complex clinical presentation and multifactorial nature of HF itself. HF patients often have comorbidities such as renal dysfunction, hepatic impairment, and frailty, which independently contribute to bleeding risk [8]. Additionally, the use of multiple medications, including anticoagulants, antiplatelet agents, and heart failure therapies such as angiotensin-converting enzyme inhibitors (ACEIs) or angiotensin receptor blockers (ARBs), further complicates risk assessment by increasing the potential for drug interactions and adverse effects [9]. Several bleeding risk assessment tools have been developed to guide clinical decision-making in anticoagulation therapy, with the HAS-BLED score being one of the most widely used [10].

The criteria of the HAS-BLED score are hypertension (uncontrolled systolic blood pressure > 160 mmHg), abnormal renal or hepatic function (one point each), stroke history (one point), bleeding history or predisposition (one point), labile international normalized ratio (INR) while on anticoagulation therapy (one point), elderly age (age > 65 years, one point), and drugs or alcohol concomitantly (one point). The minimum score for the HAS-BLED score is 0, indicating low risk, while the maximum score is 9, indicating high risk.

However, while the HAS-BLED score has demonstrated utility in predicting bleeding risk in anticoagulated patients, its applicability to the HF population is limited by several factors [11].

Firstly, many of the components of the HAS-BLED score, such as renal dysfunction and labile INR, are already prevalent in HF patients due to the underlying pathophysiology of the disease. Therefore, the discriminatory ability of the HAS-BLED score may be diminished in this population, as these factors may not accurately reflect additional bleeding risk conferred by anticoagulation therapy. Furthermore, HF-specific factors such as fluid retention, anemia, and impaired hemodynamics may contribute to bleeding risk independently of traditional risk factors captured by the HAS-BLED score. Given these limitations, a one-size-fits-all approach to bleeding risk assessment in HF patients may not be appropriate. Instead, a comprehensive evaluation that considers individual patient characteristics, including comorbidities, concomitant medications, frailty, and functional status, is essential. Incorporating patient-specific factors into the decision-making process allows for a more nuanced assessment of bleeding risk, and facilitates the development of individualized treatment plans that balance the potential benefits of anticoagulation against the risk of bleeding complications.

Anticoagulation therapy

Anticoagulation therapy in HF patients is primarily aimed at reducing the risk of thromboembolic events, particularly in those with concomitant AF or other thrombotic risk factors. The rationale for anticoagulation stems from the prothrombotic state observed in HF, which arises from a combination of factors such as impaired cardiac function, endothelial dysfunction, activation of coagulation pathways, and hemodynamic disturbances. These factors contribute to an increased risk of thrombus formation, particularly in the atria, leading to thromboembolic complications such as stroke and systemic embolism [2,12].

Several clinical trials and observational studies have evaluated the efficacy and safety of anticoagulants in HF populations. The WARCEF (Warfarin Versus Aspirin in Reduced Cardiac Ejection Fraction) trial compared warfarin and aspirin in HF patients with reduced ejection fraction (HFrEF) and sinus rhythm [13]. While warfarin did not significantly reduce the primary outcome of ischemic stroke, it was associated with a lower rate of ischemic stroke or death from any cause compared to aspirin. However, warfarin was also associated with a higher risk of major bleeding, highlighting the need for careful consideration of bleeding risk when initiating anticoagulation therapy in HF patients. In AF patients with HF, the ARISTOTLE (Apixaban for Reduction in Stroke and Other Thromboembolic Events in Atrial Fibrillation) trial demonstrated the efficacy and safety of the direct oral anticoagulant (DOAC) apixaban compared to warfarin in reducing the risk of stroke or systemic embolism [14]. Subgroup analyses of HF patients in the trial showed consistent benefits of apixaban over warfarin in terms of stroke prevention with a lower risk of major bleeding, supporting the use of DOACs as preferred anticoagulants in this population.

The role of anticoagulation in specific subsets of HF patients, such as those with reduced ejection fraction (HFrEF) or preserved ejection fraction (HFpEF), remains an area of ongoing investigation. While anticoagulation therapy is generally indicated in HF patients with AF or other thrombotic risk factors, its utility in HF patients without AF or with HFpEF is less well-established. Subanalyses of clinical trials suggest potential benefits of anticoagulation in certain high-risk HF subgroups, such as those with previous thromboembolic events or intracardiac thrombi [2,12]. However, further research is needed to delineate the optimal anticoagulation strategy in these populations and to identify patients who are most likely to benefit from therapy while minimizing the risk of bleeding complications.

In conclusion, anticoagulation therapy plays a crucial role in the management of HF patients with AF or other thrombotic risk factors by reducing the risk of thromboembolic events. While DOACs have emerged as preferred agents over warfarin due to their improved efficacy and safety profiles, the decision to initiate anticoagulation in HF patients should be individualized based on careful consideration of thrombotic and bleeding risks, as well as patient-specific factors and preferences. Ongoing research efforts are needed to further elucidate the role of anticoagulation in specific subsets.

Balancing risks and benefits

Balancing the risks of thromboembolism and bleeding poses significant challenges in the management of HF patients. HF patients are often at increased risk of both thrombotic and bleeding events due to the complex interplay of underlying pathophysiological mechanisms, comorbidities, and concomitant medications [2,10]. The decision to initiate anticoagulation therapy in HF patients requires careful consideration of individual patient characteristics, including thrombotic and bleeding risks, as well as patient preferences and values [15]. Thromboembolic events, such as stroke and systemic embolism, are major causes of morbidity and mortality in HF patients, particularly those with concomitant AF or other thrombotic risk factors [16]. Anticoagulation therapy is effective in reducing the risk of thromboembolic events and is recommended in certain HF patients based on established guidelines. However, the benefits of anticoagulation must be weighed against the risk of bleeding complications, which can have serious consequences, including worsening HF symptoms, hospitalization, and mortality [17,18].

Shared decision-making between clinicians and patients is paramount in determining the appropriateness of anticoagulation therapy in HF patients. Clinicians must engage patients in informed discussions about the potential benefits and risks of anticoagulation, taking into account individual patient preferences, values, and goals of care. Shared decision-making empowers patients to actively participate in treatment decisions, leading to greater satisfaction with care and improved adherence to therapy. To minimize bleeding risk while optimizing thromboprophylaxis in HF patients, several strategies can be employed. Firstly, a thorough assessment of bleeding risk using validated risk assessment tools, such as the HAS-BLED score, can help identify patients at higher risk of bleeding complications [19]. Clinicians should also regularly monitor patients for signs of bleeding and adjust anticoagulation therapy as needed based on changes in clinical status, laboratory parameters, and medication adherence.

Additionally, the selection of anticoagulants and dosing regimens should be individualized based on patient-specific factors, including renal function, hepatic function, and concomitant medications [20]. DOACs may offer advantages over warfarin in HF patients due to their predictable pharmacokinetics, reduced drug interactions, and lower risk of intracranial bleeding [21]. However, careful consideration should be given to dosing adjustments and monitoring requirements, particularly in patients with renal impairment or other comorbidities. Moreover, lifestyle modifications, such as dietary modifications to minimize interactions with anticoagulants and avoidance of activities that increase bleeding risk, should be discussed with patients. Finally, patient education and counseling on the signs and symptoms of bleeding, adherence to therapy, and the importance of regular follow-up are essential components of bleeding risk management in HF patients receiving anticoagulation therapy [22].

Novel anticoagulant agents and future perspectives

Emerging anticoagulant agents hold promise for improving the management of HF patients by offering alternative options with potentially favorable efficacy and safety profiles compared to traditional anticoagulants. These novel agents target specific components of the coagulation cascade and exhibit characteristics such as rapid onset of action, predictable pharmacokinetics, and reduced monitoring requirements, which may address some of the limitations associated with current therapies. One class of novel anticoagulant agents that has garnered attention in HF management is the DOACs. DOACs, including dabigatran, rivaroxaban, apixaban, and edoxaban, selectively inhibit key factors in the coagulation cascade, such as thrombin or factor Xa, thereby preventing thrombus formation [23-25]. Clinical trials evaluating the efficacy and safety of DOACs in HF patients with AF have demonstrated comparable or superior efficacy in reducing stroke and systemic embolism compared to warfarin, with lower rates of intracranial bleeding [26,27]. Furthermore, novel anticoagulant agents targeting alternative pathways in the coagulation cascade, such as factor XI inhibitors, are currently under investigation and may offer additional therapeutic options for HF patients [28,29]. These agents have the potential to provide effective thromboprophylaxis while minimizing bleeding risk, particularly in high-risk patient populations where traditional anticoagulants may be less tolerated or contraindicated [23-27].

Despite the promising efficacy and safety profiles of novel anticoagulant agents, several challenges and future directions warrant consideration. Firstly, the optimal selection and dosing of anticoagulants in HF patients require careful assessment of individual patient characteristics, including thrombotic and bleeding risks, renal function, and concomitant medications. Additionally, the integration of novel anticoagulant agents into clinical practice necessitates ongoing education and training for healthcare providers to ensure safe and effective use. Future research efforts should focus on expanding our understanding of the pathophysiological mechanisms underlying thrombosis and bleeding in HF patients, as well as identifying novel therapeutic targets for anticoagulation therapy. Furthermore, large-scale prospective studies evaluating the long-term efficacy and safety of novel anticoagulant agents in diverse HF populations are needed to inform evidence-based practice and guideline recommendations.

## Conclusions

The decision to initiate anticoagulation therapy in HF patients with increased bleeding risk presents a complex clinical challenge. While anticoagulation can reduce the risk of thromboembolic events, it carries the potential for bleeding complications that may exacerbate HF symptoms and lead to adverse outcomes. Careful assessment of individual patient factors, including thrombotic and bleeding risks, comorbidities, concomitant medications, and patient preferences, is essential for developing an optimal treatment strategy. Emerging anticoagulant agents, such as DOACs, offer promising alternatives to traditional therapies like warfarin. DOACs have demonstrated comparable or superior efficacy in stroke prevention, with a lower risk of intracranial bleeding in certain patient populations. However, their use in HF patients with increased bleeding risk requires careful consideration of dosing adjustments and monitoring requirements. Ultimately, optimizing thromboprophylaxis while minimizing bleeding risk in HF patients necessitates a multidisciplinary approach that incorporates shared decision-making, comprehensive risk assessment, individualized treatment strategies, and patient education. By addressing the paradoxical relationship between HF, increased bleeding risk, and the potential benefits of anticoagulation, clinicians can improve patient outcomes and enhance the quality of life for this vulnerable population.
